# Ferric carboxymaltose in reducing blood transfusions and infections after cardiac surgery

**DOI:** 10.1016/j.xjon.2024.09.009

**Published:** 2024-09-19

**Authors:** Tuomas O. Kiviniemi, Vesa Anttila, Kristiina Pälve, Marko Vesanen, Joonas Lehto, Markus Malmberg, Tuija Vasankari, K.E.Juhani Airaksinen, Jarmo Gunn

**Affiliations:** a Heart Center, Turku University Hospital, Turku, Finland; b Department of Clinical Medicine, University of Turku, Turku, Finland; c Department of Surgery, University of Turku, Turku, Finland; d Medicine, Turku University Hospital, Turku, Finland

**Keywords:** coronary artery bypass surgery, aortic valve replacement, mitral valve surgery, iron supplementation

## Abstract

**Objective:**

Iron supplementation may reduce postoperative anemia, blood transfusions, and infections in patients undergoing surgery. We sought to assess efficacy and safety of prophylactic intravenous iron supplementation in patients without anemia undergoing cardiac surgery.

**Methods:**

In this investigator-initiated industry-sponsored single-center randomized double-blind parallel group trial, we enrolled patients undergoing coronary bypass, aortic or mitral valve or ascending aortic surgery who fulfilled prespecified iron blood test safety criteria. Patients were randomized to receive either a single intravenous 1000 mg dose of ferric carboxymaltose (FCM) or placebo (saline only). Independent unblinded study nurse administered the infusion with masked lines and cannula 2 to 21 days before surgery. Primary efficacy end point was a composite of in-hospital blood transfusions >2 U and nosocomial infection. The trial was registered in Eudract (2017-004901-41).

**Results:**

Altogether 171 patients were screened and 78 randomly assigned to FCM (n = 39) or placebo (n = 39). Trial was prematurely discontinued for futility with regard to reaching the primary end point by the recommendation of the independent data monitoring committee. The primary end point occurred in 3 (7.7%) versus 3 (7.7%) (*P* = 1.00) of patients assigned to FCM and placebo, respectively, with no difference in blood transfusions >2 U. Fewer hospital readmissions by 3 months follow-up (1 [2.6%] vs 8 [20.5%]; *P* = .028) were noted in FCM group in a post hoc analysis. Ferritin levels were higher in the FCM group at 3 months indicating more preserved iron stores.

**Conclusions:**

Prophylactic treatment with FCM was safe but did not reduce the need for blood transfusions or postoperative infections at index hospitalization in patients without anemia undergoing cardiac surgery.

**Figure:**
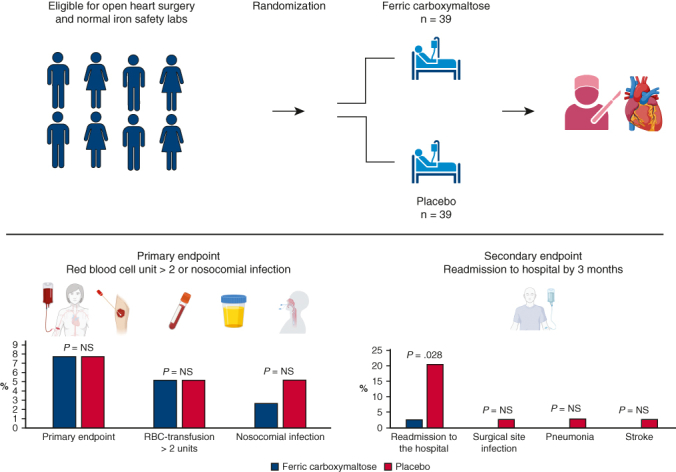
Main results of the trial.

**Central Message**

Ferric carboxymaltose use was safe, but did not reduce red blood cell need or infections compared with placebo at index hospitalization.

**Perspective**

Larger randomized trials are needed to assess whether preoperative intravenous iron supplementation decreases hospital readmissions and decompensated heart failure after cardiac surgery.

Cardiac surgery is associated with significant blood loss, often leading to anemia and need for blood transfusions. Anemia is a well-known predictor of adverse outcomes, including increased mortality; longer hospital stays; and higher rates of postoperative complications, including infections.^1^, ^2^, ^3^, ^4^ Red blood cell transfusions (RBC) are frequently needed to treat anemia, but nearly half of patients may remain anemic at 50 days after surgery.^5^ Furthermore, use of RBCs during cardiac surgery is associated with its own set of risks and complications, such as transfusion reactions, infections, thromboembolic complications, early and long-term mortality, and increased health care costs.^4^, ^5^, ^6^, ^7^

Intravenous iron supplementation has emerged as an appealing strategy to reduce the incidence of postoperative anemia and the need for blood transfusions in patients undergoing surgery. Preoperative iron supplementation led to a 50% reduction in postoperative nosocomial infection rates in patients undergoing orthopedic surgery.^8^^,^^9^ However, the effects of preoperative intravenous iron supplementation on postoperative blood transfusion requirement and infections are still unclear in patients without anemia undergoing cardiac surgery.

In this randomized trial, we aimed to investigate the efficacy and safety of preoperative ferric carboxymaltose in reducing the incidence of postoperative need for blood transfusions and postoperative infections in patients without anemia undergoing cardiac surgery.

## Methods

### Study Design and Participants

A Phase IV Double-blind, Randomised, Parallel Group Comparison of the Efficacy and Safety of Preoperative Intravenous Ferric Carboxymaltose and Placebo in the Treatment of Patients Undergoing Elective or Urgent Cardiac Surgery (PREFER-CPB) was an investigator-initiated, industry-sponsored single-center double-blinded, randomized, parallel group comparison in 1:1 ratio to evaluate the efficacy of ferric carboxymaltose over placebo on reducing the 90-day incidence of postoperative blood transfusions and nosocomial infections in patients undergoing elective or urgent cardiac surgery. The original study protocol as well as an extended Methods sections are in Appendix E1. The trial was approved by the Ethics Committee of Southwestern Finland Hospital District (approval ID: ETMK 128/1800/2017; referred by the National Committee on Medical Research and Ethics, Tukija) and the Finnish Medicines Agency (approval ID: KLNRO 33/2018). The study was registered at Eudract: 2017-004901-41.

Patients referred for elective or urgent cardiac surgery on cardiopulmonary bypass were identified and screened. All included patients presented with cardiac disease requiring surgery and open cardiac surgery was considered the appropriate treatment strategy according to the current guidelines.^10^, ^11^, ^12^, ^13^, ^14^ Exclusion criteria included age younger than 35 years, emergency or salvage surgery (<24 hours from decision to operate), participation in another clinical study or treatment with another investigational product 30 days before randomization, moribund patient not expected to survive surgery, active malignant disease with a short life expectancy (eg, malignant cancer, hemoglobin >155 g/dL for women and >167 g/dL for men), ferritin >150 μg/L for women and >400 μg/L for men; dialysis therapy for chronic renal failure; hemochromatosis; polycytemia vera; known allergic reaction linked to iron medication; an ongoing oral iron therapy at the time of randomization or iron infusion given 0 to 30 days before randomization; any other condition or therapy, which in the investigators’ opinion would make the subject unsuitable for this study and will not allow active participation for the full planned study period.

Patients without exclusion criteria were screened and evaluated by the researchers and all gave written informed consent for publication. Before randomization, baseline laboratory values were measured and patients exceeding prespecified iron safety values were excluded. Patients were randomized to either a single dose of 1000 mg intravenous ferric carboxymaltose in 100 mL normal saline; or placebo (100 mL saline only) 48 hours to 21 days (504 hours) preoperatively. To blind the patient and researchers, an unblinded study nurse independent of the research personnel administered the infusion with intravenous lines and cannulae covered in drapes. Patients were monitored for 30 minutes for adverse events or hypersensitivity. All members of the study group and hospital personnel were unaware of the treatment allocation.

### Outcomes

The primary end point was a composite of blood transfusions >2 U packed RBC or postoperative infection during the index hospitalization. Postoperative infection was defined by the Centers for Disease Control and Prevention protocol (https://www.cdc.gov/hai/ssi/ssi.html) and surgeons were trained before study commencement on the criteria to achieve uniform reporting of infections (see Appendix E1 for details).

Secondary end points were RBC transfusion rates; postoperative nosocomial infection rates; length of stay (intensive care unit and total); 90 days and 1-year mortality; readmissions due to surgical site infection (mediastinitis, deep wound, and vein harvesting site infection) or postpericardiotomy syndrome up to 1 year; major adverse cardiac and cerebrovascular events, including nonfatal new onset myocardial infarction unrelated to surgery, cardiovascular death, stroke, transient ischemic attack within 90 days; new-onset postoperative atrial fibrillation (90 days); and postdischarge need for oral or intravenous iron therapy at 3 months.

Secondary biomarker end points were discharge hemoglobin value, RBC and iron indexes, RBC and iron indexes at 3 months follow-up, and left ventricular ejection fraction at 3 months follow-up. Quality of life was assessed between baseline, 90 days, and 1 year using EuroQOL 5-D. Clinically significant treatment emergency adverse events were collected during hospitalization from vital signs data, laboratory data, physical examinations, and spontaneous reporting when conscious. World Health Organization definition of anemia (130 g/L for men and 120 g/L for women) was used.

### Adjudication

Data were monitored by a third party. Primary events were adjudicated by 2 independent experts (1 cardiac surgeon and 1 cardiac anesthesiologist) after study completion.

### Data Monitoring Committee

The independent data monitoring committee consisted of a cardiac surgeon, a clinical pharmacologist, a hematologist, and a biostatistician.

### Statistical Analysis

Data were analyzed using SAS (SAS Institute Inc) and R (R Foundation for Statistical Computing), and graphs were drawn using Biorender and GraphPad Prism. Continuous variables were reported as mean ± SD if normally distributed and as median (interquartile range) if skewed. The data were tested for normal distribution using the Shapiro-Wilk test. Categorical variables were described as counts and percentages. Unpaired *t* test and Mann-Whitney test were used for univariable analysis.

### Role of the Funding Entity

The study sponsor Vifor Pharma had no role in the study design, decision to amend the protocol to include patients other than those undergoing coronary artery bypass grafting; the collection, analysis, and interpretation of data; the writing of the report; or in the decision to submit the manuscript for publication. The authors had full access to all the data in the study and had final responsibility for the decision to submit for publication.

## Results

**Figure 1:**
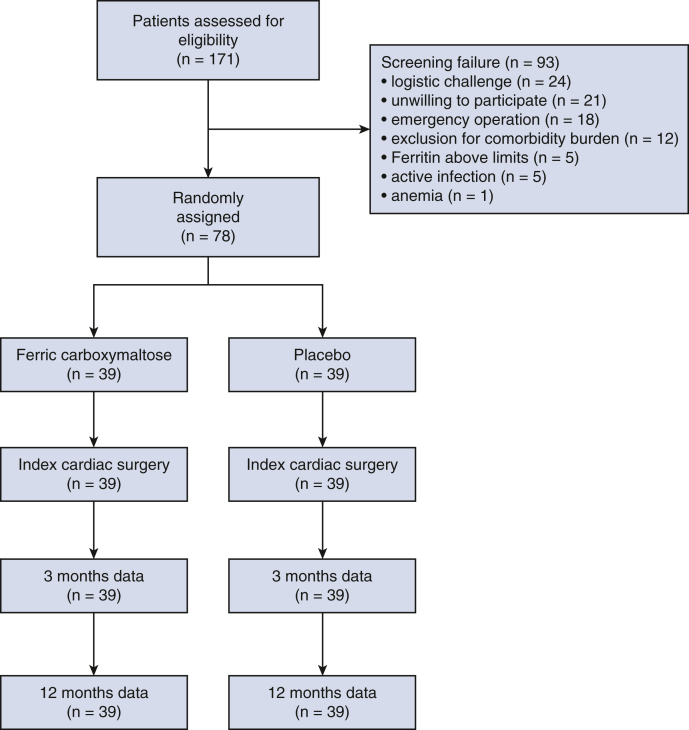
Flow chart of the PREFER-CBP trial. *PREFER-CBP*, Preoperative Intravenous Ferric Carboxymaltose in the Treatment of Patients Undergoing Elective or Urgent Cardiac Surgery.

Between September 2018 and February 2022, altogether 171 patients were screened and 78 randomly assigned to ferric carboxymaltose (n = 39) and placebo (n = 39) (Figure 1). Study medicinal product was infused median 5 days (range, 3-14 days) before index operation. All participants in both groups received their randomly allocated treatment. No infusion-related serious adverse events were noted in either group. The trial was prematurely terminated for futility by the recommendation of the independent data monitoring committee after prespecified interim analysis of 50 patients with complete 12 months’ follow-up. The recommendation was based on the likelihood of not reaching the primary end point with the anticipated enrollment of 210 patients. Nevertheless, there were no safety issues with the medicinal product. Follow-up was complete in all 78 patients who were enrolled and randomized by the time of the recommendation.

Baseline characteristics were similar between the groups and detailed in Table 1. Share of patients undergoing coronary bypass was 58.3%, aortic valve replacement 17.9%, mitral valve surgery 16.7%, and ascending aortic surgery 17.9%. Perioperative variables are presented in Table 2. There were no significant differences between the groups in indications for surgery, urgency, operation-related variables or intensive care-related variables. Mean hemoglobin levels preoperatively were similar (142 ± 12.9 vs 145 ± 12.4 g/L for ferric carboxymaltose vs placebo, respectively) (Table 3).

**Table 1:** Baseline demographic characteristics of patients undergoing cardiac surgery treated preoperatively with intravenous ferric carboxymaltose versus controls

Variable	Ferric carboxymaltose (n = 39)	Placebo (n = 39)	*P* value
Male sex	33 (84.6)	35 (89.7)	.737
Age (y)	67.2 ± 9.2	64.7 ± 7.6	.201
Smoking status (%)			.188
No	26 (66.7)	21 (53.8)	
Yes	0 (0.0)	3 (7.7)	
Exsmoker	13 (33.3)	15 (38.5)	
Alcohol consumption			.192
Abstinent	10 (26.3)	4 (10.3)	
Moderate use	27 (71.1)	33 (84.6)	
Heavy use∗	1 (2.6)	2 (5.1)	
NYHA functional class (%)			.610
I	10 (30.3)	11 (34.4)	
II	17 (51.5)	15 (46.9)	
III	6 (18.2)	4 (12.5)	
IV	0 (0.0)	2 (6.2)	
CCS class (%)			.920
0	5 (17.9)	7 (23.3)	
I	7 (25.0)	8 (26.7)	
II	11 (39.3)	12 (40.0)	
III	4 (14.3)	2 (6.7)	
IV	1 (3.6)	1 (3.3)	
Body mass index	27.8 (25.3-32.0)	28.6 (24.4-31.6)	.956
Treatment for hypertension	23 (59.0)	24 (61.5)	1.000
Hypercholesterolemia	18 (46.2)	18 (46.2)	1.000
Diabetes	9 (23.1)	11 (28.2)	.796
History of atrial fibrillation	6 (15.4)	6 (15.4)	1.000
Congestive heart failure	2 (5.1)	3 (7.7)	1.000
History of myocardial infarction	0 (0.0)	2 (5.1)	.494
Comorbidities			
Chronic lung disease	7 (17.9)	5 (12.8)	.754
History of stroke	2 (5.1)	2 (5.1)	1.000
History of TIA	1 (2.6)	2 (5.1)	1.000
Renal insufficiency	1 (2.6)	0 (0.0)	1.000
Preoperative medication			
Acetylsalicylic acid	20 (51.3)	21 (53.8)	1.000
Clopidogrel	5 (12.8)	0 (0.0)	.055
Other P2Y12 inhibitor	1 (2.6)	0 (0.0)	1.000
Warfarin	3 (7.7)	0 (0.0)	.240
Direct oral anticoagulant	2 (5.1)	4 (10.3)	.675
Beta blocker	22 (56.4)	23 (59.0)	1.000
Statin	30 (76.9)	25 (64.1)	.321
ACE inhibitor	9 (23.1)	17 (43.6)	.093
AT2-receptor blocker	14 (35.9)	8 (20.5)	.208
Corticosteroid	0 (0.0)	1 (2.6)	1.000
Nonsteroidal anti-inflammatory agents	0 (0.0)	1 (2.6)	1.000
Proton pump inhibitors	8 (20.5)	3 (7.7)	.193
Antidepressant†	1 (2.6)	0 (0.0)	1.000

Values are presented as n (%) for categorical variables and mean ± SD or median (interquartile range) for continuous variables as appropriate. *NYHA*, New York Heart Association classification; *CCS*, Canadian Cardiovascular Society angina grade; *TIA*, transient ischemic attack; *ACE*, angiotensin-converting enzyme; *AT2*, angiotensin 2.

∗ Heavy alcohol use defined as 24 weekly doses and >16 doses for women.

† Selective serotonin reuptake inhibitor.

**Table 2:** Perioperative characteristics of patients undergoing cardiac surgery treated preoperatively with intravenous ferric carboxymaltose versus placebo

Variable	Ferric carboxymaltose (n = 39)	Placebo (n = 39)	*P* value
Surgical procedure			
Coronary artery bypass	25 (64.1)	20 (51.3)	.359
Aortic valve replacement	7 (17.9)	7 (17.9)	1.000
Mitral valve replacement	5 (12.8)	0 (0.0)	.055
Mitral valvuloplasty	4 (10.3)	4 (10.3)	1.000
Ascending aortic surgery	5 (12.8)	9 (23.1)	.376
Urgent procedure	4 (10.3)	7 (17.9)	.347
Preoperative LVEF (%)	60.0 (54.5-63.5)	57.5 (50.5-61.7)	.503
Preoperative Hb (g/L)	142 ± 12.8	146 ± 12.4	.23
ICU length of stay (d)	0.94 (0.87-1.04)	0.94 (0.91-1.02)	.669
Total fluids, first 12 h (mL)	2249 (1877-2841)	2433 (2040-3099)	.433
Chest tube output, first 12 h (mL)	380 (305-600)	403 (300-528)	.883
Diuresis in the first 12 h (mL)	1240 (1023-1558)	1355 (993-1611)	.551
Time on ventilator (h)	10.4 (9.0-18.2)	10.6 (9.4-12.4)	.870
Time on vasoactive medication (h)	22.2 (10.8-26.9)	18.1 (10.1-23.4)	.323

Values are presented as n (%) for categorical variables and mean ± SD or median (interquartile range) for continuous variables. *LVEF*, Left ventricular ejection fraction; *Hb*, hemoglobin; *ICU*, intensive care unit.

**Table 3:** Biochemical data preoperatively and postoperatively (min/max values) and at 90-day follow-up

Variable	Ferric carboxymaltose (n = 39)	Placebo (n = 39)	*P* value
Preoperative			
B-Hb (g/L)	142.15 ± 12.88	145.64 ± 12.36	.226
B-Eryt (E12/L)	4.67 ± 0.45	4.78 ± 0.47	.297
B-Hkr (ratio)	0.43 ± 0.04	0.44 ± 0.03	.346
E-MCV (fl)	92.00 (89.00-93.00)	91.00 (89.00-93.50)	.736
E-MCH (pg)	30.00 (30.00-31.50)	31.00 (29.00-32.00)	.772
B-Trom	223.00 (184.500-259.50)	228.00 (190.50-254.50)	.822
Transferrin Fe-sat%	25.65 ± 8.99	26.70 ± 6.39	.559
fP-Fe	16.00 (12.50-18.50)	16.50 (14.25-19.00)	.396
fP-Transf	2.51 ± 0.31	2.56 ± 0.37	.524
P-Ferrit	131.00 (79.00-221.00)	151.50 (88.75-243.75)	.490
P-TfR	2.80 (2.50-3.32)	2.85 (2.30-3.40)	.928
P-B12 vitamin	372.50 (293.25-472.50)	440.00 (310.00-523.00)	.309
P-folic acid	486.00 (287.00-1372.00)	367.00 (227.00-1748.75)	.843
Postoperative min/max			
B-Hb (g/L) min	98.67 ± 14.34	100.45 ± 15.09	.597
B-Hkr (ratio) min	0.30 ± 0.04	0.30 ± 0.04	.618
B-Eryt (E12/L) min	3.21 ± 0.50	3.28 ± 0.51	.592
E-MCV (fl) min	91.23 ± 3.43	91.08 ± 3.23	.842
E-MCH (pg) min	31.00 (29.50-31.00)	31.00 (30.00-31.00)	.879
B-Trom min	131.69 ± 45.46	133.95 ± 42.86	.823
90-d follow-up			
B-Hb (g/L)	135.83 ± 12.31	130.68 ± 15.41	.118
B-Hkr (ratio)	0.42 ± 0.03	0.41 ± 0.05	.277
B-Eryt (E12/L)	4.53 ± 0.47	4.55 ± 0.57	.913
E-MCV (fl)	92.75 ± 3.48	89.89 ± 3.74	.001
E-MCH (pg)	30.00 (29.00-31.00)	29.00 (28.00-30.00)	.002
B-Leuk (E9/l)	7.06 ± 1.67	6.87 ± 1.61	.632
B-Trom	229.00 (198.25-291.25)	254.50 (218.25-283.75)	.364
Transferrin Fe-sat%	21.69 ± 7.99	17.06 ± 5.36	.009
fP-Fe	12.12 ± 4.31	11.55 ± 3.65	.569
fP-Transf	2.24 ± 0.28	2.74 ± 0.49	<.001
P-Ferrit	310.00 (204.50-398.00)	68.00 (38.50-129.25)	<.001
P-TfR	4.00 (2.95-4.85)	4.35 (3.58-5.10)	.167
P-B12 vitamin	364.00 (289.00-424.00)	408.00 (299.75-488.75)	.179
P-folic acid	1054.00 (352.00-1753.00)	418.00 (137.40-1710.00)	.297

Values are presented as mean ± SD or median (interquartile range) for continuous variables as appropriate. *B*, Blood; *Hb*, hemoglobin; *Eryt*, erythrocytes; *Hkr*, hematocrit; *E*, erythrocyte; *MCV*, mean corpuscular volume; *MCH*, mean corpuscular hemoglobin; *Trom*, thrombocytes; *Transferrin Fe-sat%*, transferrin iron saturation; *fP*, fasting plasma; *Fe*, iron; *Transf*, transferrin; *Ferrit*, ferritin; *TfR*, transferrin receptor; *Leuk*, leukocytes.

**Figure 2:**
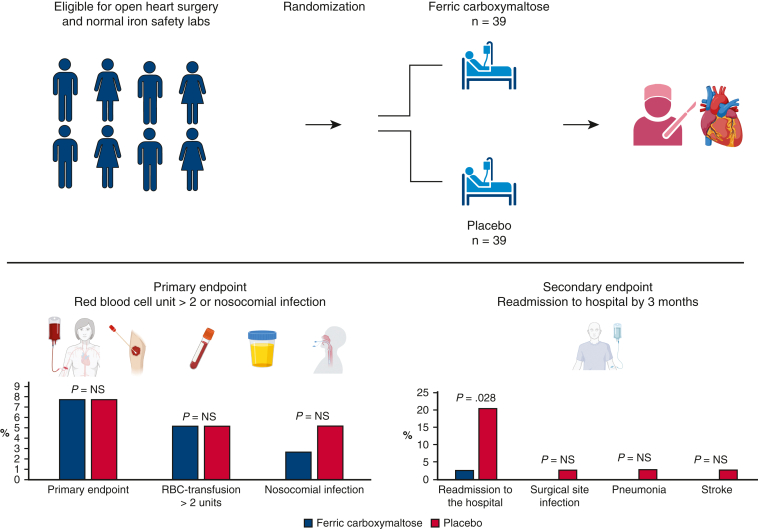
Altogether, 78 patients were randomly assigned to intravenous ferric carboxymaltose (n = 39) or placebo (n = 39) groups. No difference was seen in the need for packed red blood cells transfusions or nosocomial infections patients assigned to ferric carboxymaltose and placebo, respectively, during index hospitalization. In a post hoc analysis, fewer hospital readmissions by 3 months were noted in ferric carboxymaltose group.

**Figure E1:**
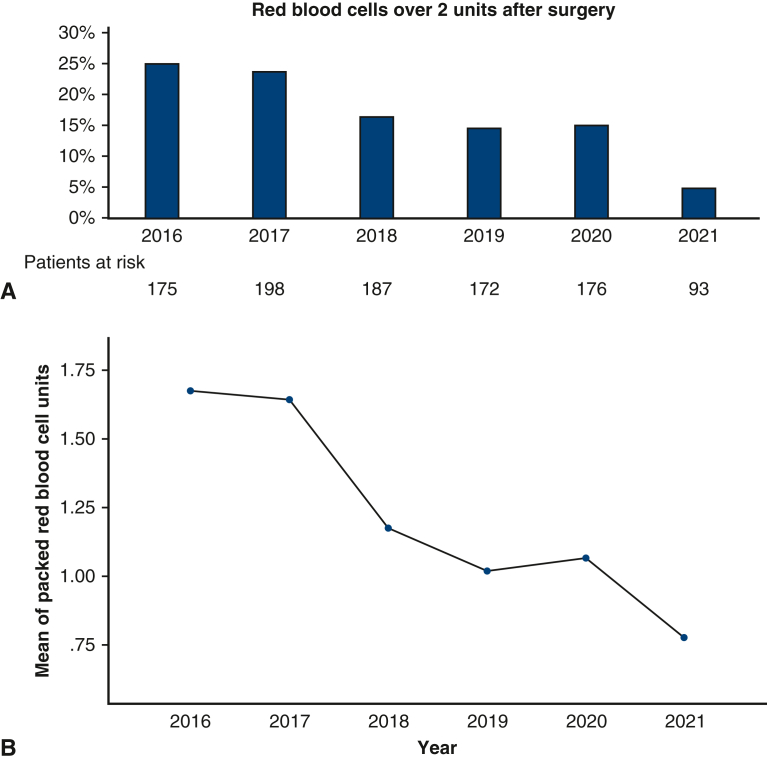
Decrease in red blood cell (*RBC*) use between 2016 and 2021 at the institution. A, The decrease in >2 U RBCs per patient use at the institution between 2016 and 2021. B, The mean of units per patient of RBCs between 2016 and 2021. Reason for lower use was the utilization of institutional protocol for more restrictive fluid resuscitation and conservative RBC use, which was taken into clinical practice at the time of trial at the hospital. This resulted in lower than expected rate of RBC use. Data derived from the prospective CAREBANK study (NCT030444259).

Figure 2 depicts the main results of the trial. Primary end point occurred in 3 (7.7%) versus 3 (7.7%) (*P* = 1.00), need for RBC transfusions 2 (5.1%) versus 2 (5.1%) (*P* = 1.00), and nosocomial infections 1 (2.6%) versus 2 (5.1%) (*P* = 1.00) in patients assigned to ferric carboxymaltose and placebo, respectively, during index hospitalization (Table 4). Share of patients who received any blood transfusion was the same (6 [15.4%] vs 7 [17.9%]; *P* = 1.00, respectively). Lower than anticipated use of RBC was likely due to the new institutional strategy of targeted blood product use that was launched at the time of trial (see Figure E1 for details).

**Table 4:** Postoperative outcomes of patients undergoing cardiac surgery treated preoperatively with intravenous ferric carboxymaltose versus placebo

Variable	Ferric carboxymaltose (n = 39)	Placebo (n = 39)	*P* value
Primary end point∗	3 (7.7)	3 (7.7)	1.000
RBC transfusion >2 U	2 (5.1)	2 (5.1)	1.000
Nosocomial infection†	1 (2.6)	2 (5.1)	1.000
Resternotomy	3 (7.7)	4 (10.3)	.422
For bleeding	2 (5.1)	4 (10.3)	.675
For tamponade	1 (2.6)	1 (5.1)	1.000
Surgical site infection, superficial†	0	1 (2.6)	1.000
Surgical site infection, deep†	0	0	1.000
Pneumonia	0	0	1.000
Mediastinitis	0	0	1.000
Postoperative hemorrhage	2 (5.1)	5 (12.8)	.430
Hemorrhage requiring operative treatment	2 (5.1)	4 (10.3)	.675
Acute myocardial infarction	1 (2.6)	0 (0.0)	1.000
Thromboembolism	0	2 (5.1)	.240
Stroke	0	1 (2.6)	1.000
New-onset atrial fibrillation	12 (30.8)	8 (20.5)	.435
Cardiovascular mortality	1 (2.6)	0	1.000
Other mortality	1 (2.6)	0	1.000
0-3 mo follow-up			
New-onset atrial fibrillation after discharge	1 (2.6)	2 (5.1)	1.000
Acute decompensated heart failure	0 (0.0)	3 (7.7)	.240
Stroke	0	1 (2.6)	1.000
Bleeding event	0	1 (2.6)	1.000
Readmission	1 (2.6)	8 (20.5)	.028
For surgical site infection	0	1 (2.6)	1.000
For pneumonia	0	1 (2.6)	1.000
For stroke	0	1 (2.6)	1.000
For rhythm disorder	0	2 (5.1)	.494
For other infection	0	1 (2.6)	1.000
For other reason	1 (2.6)	2 (5.1)	1.000
Cardiovascular mortality	0	0	1.000
All-cause mortality	0	0	1.000
3-12 mo follow-up			
New-onset atrial fibrillation after discharge	0	0	1.000
Acute decompensated heart failure	0	0	1.000
Stroke	0	0	1.000
Bleeding event	0	0	1.000
Readmission to the hospital	5 (12.8)	5 (12.8)	1.000
For surgical site infection	0	0	1.000
For pneumonia	0	2 (5.1)	.494
For other infection	3 (7.7)	5 (12.8)	.71
For stroke	0	0	1.000
For rhythm disorder	0	0	1.000
For other reason	2 (5.1)	1 (2.6)	1.000
Cardiovascular mortality	0	0	1.000
All-cause mortality	0	1 (2.6)	1.000

Values are presented as n (%). *RBC*, Packed red blood cells.

∗ Primary end point is composite of postoperative nosocomial infection or red blood cell transfusion >2 U during index hospitalization.

† Infections registered according to Centers for Disease Control and Prevention criteria.

In a post hoc analysis, readmission to hospital was less frequent (1 [2.6%] vs 8 [20.5%]; *P* = .028) at 3 months in ferric carboxymaltose group. At 3- and 12-months’ follow-up, the incidences of other secondary end points (cardiovascular death, noncardiovascular death, stroke, myocardial infarction, and new-onset atrial fibrillation after hospital discharge) were similar between the groups. No differences in adverse events by any organ class were noted (14 [35.9%] vs 17 [43.6%]).

**Figure 3:**
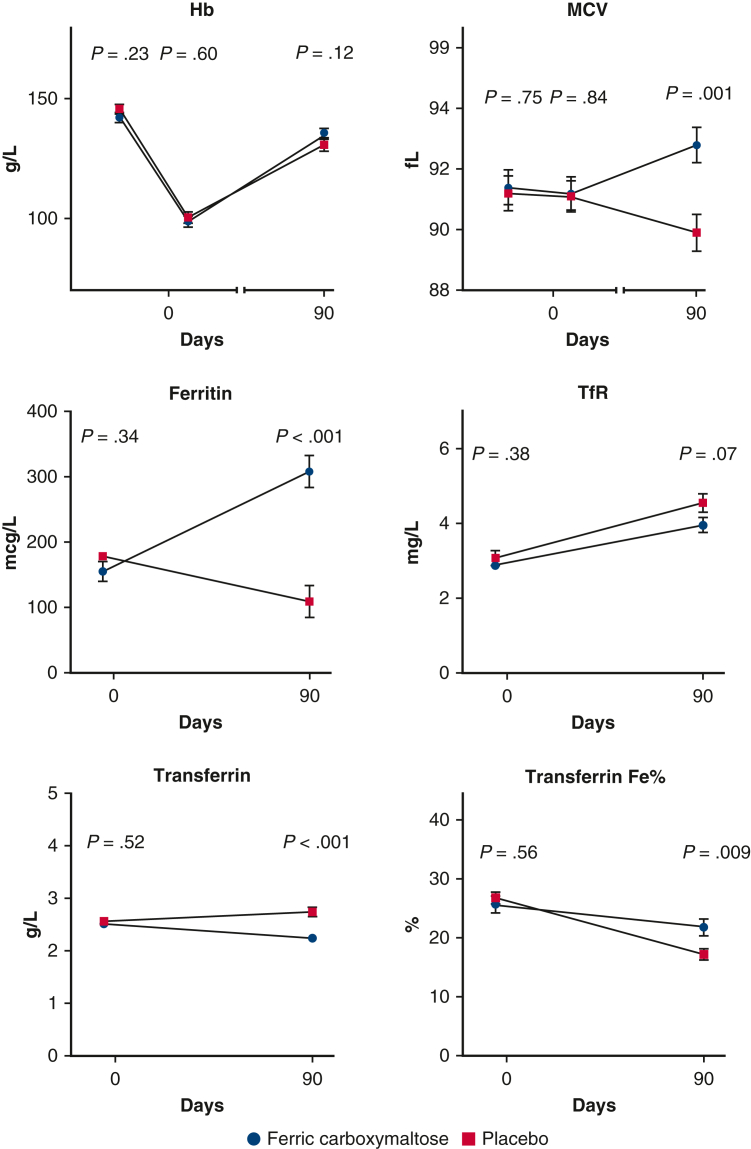
*Upper panels* show hemoglobin (*Hb*) and mean corpuscular volume (*MCV*) preoperatively, lowest at index hospitalization and at 3 months follow-up; and middle and lower panels show ferritin, transferritin reception (*TfR*), transferrin, and transferrin iron (*Fe*) % levels preoperatively and at 3-months follow-up in patients treated with ferric carboxymaltose (*circle*) versus placebo (*square*). Day 0 indicates index operation day.

**Figure E2:**
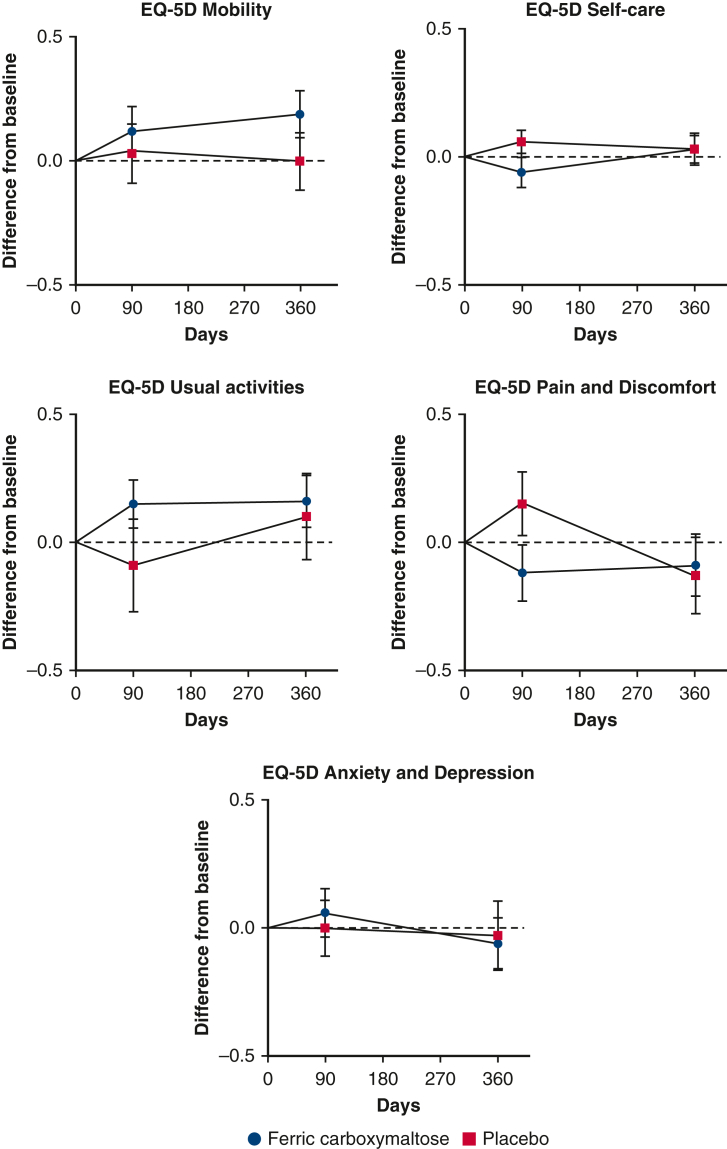
Change in EQ-5D quality-of-life measures. No significant differences were observed between ferric carboxymaltose versus placebo groups at 3 and 12 months of follow-ups.

No statistically significant differences were observed in EuroQOL 5-D quality-of-life questionnaires before operation or at 3- and 12-months’ follow-up (Figure E2). There were no differences between the study groups in any component of blood count, serum creatinine level, or troponin T level before surgery or during the index hospitalization (Table 3). Postoperative hemoglobin levels were similar in the groups during index hospitalization and at 3-months’ follow-up (Figure 3). There were no differences in the proportion of anemic (World Health Organization criteria) patients at 3 months (9 [23.1%] vs 14 [35.9%]; *P* = .16) or at 12 months (0 vs 0) in ferric carboxymaltose and placebo groups, respectively. However, mean corpuscular volume, ferritin, and transferrin were higher—and transferrin receptor levels were lower—in the ferric carboxymaltose group compared with the placebo group at 3 months' follow-up indicating increased iron stores (Figure 3). As anticipated, Pi levels were lower in the ferric carboxymaltose group compared with placebo during index hospitalization, but no longer at 3 months’ follow-up.

## Discussion

The findings of this trial indicate that prophylactic preoperative intravenous iron supplementation in patients undergoing cardiac surgery is safe, but may not be effective in reducing the need for postoperative blood transfusions or the incidence of infections during hospital stay. Although prior studies suggest that intravenous iron may reduce the incidence of anemia and the need for blood transfusions in patients undergoing other types of surgery, our trial did not demonstrate these benefits in cardiac surgery. In a hypothesis-generating post hoc analysis; however, there was a significant reduction in hospital readmissions by 3 months follow-up.

More than one-third of patients undergoing any type of open cardiac surgery receive blood transfusions.^4^^,^^5^^,^^15^^,^^16^ Based on this, we hypothesized that the need for blood transfusions could be lowered by filling the iron storage upfront before surgery in patients who are not anemic. Reasons for the need of blood transfusions include blood loss related to surgery itself, use of cardiopulmonary bypass, dilution related to fluid resuscitation with crystalloids, and major surgical site bleed postoperatively. Lack of clinically meaningful beneficial effects with prophylactic iron supplementation after cardiac surgery may be due to many factors. First, the operation-related blood loss is in many patients too large to be compensated by restoring or filling the iron deposits. Secondly, the use of RBC decreased between 2016 and 2020 significantly because of more restrictive fluid resuscitation and conservative RBC use protocol, which was taken into clinical practice at the time of trial at the hospital. This resulted in lower than expected rate of RBC use—and theoretically affected the infection rates (see Figure E1). Thirdly, the optimal dosing and timing of intravenous iron supplementation in patients undergoing cardiac surgery remain unclear. Our study used a standardized single infusion protocol for intravenous iron administration, but individual patient needs may vary based on factors such as baseline hemoglobin levels, comorbidities, and the extent of surgical bleeding, although ferritin levels were significantly higher 3 months after surgery in patients treated with ferric carboxymaltose. Further research is needed on preoperatively anemic patients who might benefit from intravenous iron supplementation and to determine the most effective dosing and timing of this intervention in patients undergoing cardiac surgery. A third potential explanation for our findings is that our sample size was not large enough to detect significant differences between the study groups.

Our post hoc analysis on less-frequent hospital readmissions in the ferric carboxymaltose group generates a hypothesis that the benefit of preoperative prophylactic iron use may become evident later than during the index hospitalization. By the 3 months’ follow-up, patients in the ferric carboxymaltose group had higher ferritin levels, indicating better iron stores. Most of the morbidity in the control group were due to endpoints such as infections and decompensated heart failure, which are plausibly related to lack of adequate iron stores. Notably, there were no hospital readmissions for decompensated heart failure nor was it observed in outpatient visits by 12 months in the treatment group. In line, in the recent Preoperative Intravenous Iron to Treat Anaemia in Major Surgery (PREVENTT) trial there was lower readmission rates at 60 and 90 days in the iron treatment arm.^17^

It is important to note that intravenous iron supplementation was generally safe and well tolerated in patients undergoing cardiac surgery. Our study did not identify any serious adverse events associated with intravenous iron administration, suggesting that it can be safely used in this patient population.

Preoperative anemia remains a significant risk factor for adverse outcomes in patients undergoing cardiac surgery.^1^, ^2^, ^3^, ^4^ Intravenous iron supplementation has been shown to effectively treat preoperative anemia in other patient populations, and it is possible that preoperatively anemic patients may benefit from this intervention although our trial did not find significant effects in patients without anemia during index hospitalization. Nevertheless, the use of ferric carboxymaltose was not superior to placebo in a large randomized trial of anemic patients before major open elective abdominal surgery.^17^

Trial has limitations that need to be taken into account when interpreting the findings. Trial was originally powered to show 35% reduction in the primary end point with the 220 patients sample size. Enrolled sample size is too low to draw other than hypothesis generating assumptions of secondary end points. Nevertheless, ferritin levels were significantly higher in ferric carboxymaltose group 3 months after surgery indicating the potential for having an effect on outcomes related to lower iron stores such as decompensated heart failure or infections. Decompensated heart failure after hospital discharge was observed in 7.7% of patients in the control group, but this did not result in hospital readmission because these patients were treated at the outpatient clinic. Trial mainly enrolled elective patients. Use of RBC and rate of infections may be higher in patients treated for acute coronary syndromes. Previously, iron in combination with erytropoetin stimulating agents, vitamin B12, and folic acid decreased the need for blood transfusions.^18^ To minimize the risk of confounding related to giving many agents simultaneously, we sought to assess the effect of a single product, easily administrable single dose, in a placebo-controlled manner.

## Conclusions

Prophylactic treatment with ferric carboxymaltose was safe but did not reduce the need for blood transfusions and postoperative infections in patients undergoing cardiac surgery in this prematurely discontinued trial. Post hoc analysis of hospital readmissions after surgery generates a potential end point for future trials in iron supplementation in surgery.

## Conflict of Interest Statement

The authors reported no conflicts of interest.

The *Journal* policy requires editors and reviewers to disclose conflicts of interest and to decline handing or reviewing manuscripts for which they may have a conflict of interest. The editors and reviewers of this article have no conflicts of interest.

## Appendix E1 Supplemental Methods

Originally, the trial included only patients undergoing coronary artery bypass grafting for stable coronary artery disease. Protocol was amended to also include other types of cardiac surgery due to slow enrollment and COVID-19 pandemic. At the time of amendment, the trial name was changed from A Phase IV Double-blind, Randomised, Parallel Group Comparison of the Efficacy and Safety of Preoperative Intravenous Ferric Carboxymaltose in the Treatment of Patients Undergoing Elective or Urgent Coronary Artery Bypass Surgery (PREFER-CABG) to A Phase IV Double-blind, Randomised, Parallel Group Comparison of the Efficacy and Safety of Preoperative Intravenous Ferric Carboxymaltose in the Treatment of Patients Undergoing Elective or Urgent Cardiac Surgery (PREFER-CBP) and sample size calculation was redone.

### Randomization and Blinding

Randomization envelopes were produced by an independent statistician using https://www.sealedenvelope.com randomization algorithm. There was a stratified block randomization (block size of 20) according to indication for surgery (coronary bypass or valve surgery). In each block 10 and 10 patients were randomized to active treatment and placebo groups, respectively. Patients were considered randomized as soon as the envelope was opened and treatment allocation given.

### Postrandomization Procedures and Follow-up

Following randomization and infusion, all patients were treated according to institutional pathways for surgery. Patients were monitored for adverse outcomes and events during index hospitalization and patient records were evaluated to identify outcomes up to 90 days. Patients were reassessed at 2 to 3 months during the routine postoperative outpatient visit and blood iron variables were measured. All patients had a follow-up telephone call from the research nurse at 12 months after index surgery.

### Sample Size Calculation

A total of 210 patients were planned to be randomized in 1:1 ratio to the active treatment and control groups. Altogether 34.7% to 41.7% of patients received ≥2 U packed red blood cells (RBC) based on Swedeheart, and a large US database, whereas the use of >2 U RBC was 13%.^15^^,^^16^^,^^19^ Local data about the rate of deep wound infections was approximately 6%.^15^ The estimated overlap of RBC in patients with postoperative nosocomial infection was 50% based on internal institutional data, and thus, the percentage of patients with postoperative nosocomial infection without RBC was 7.5%.^4^^,^^15^^,^^16^ Based on previously documented 30% to 60% risk reduction with intravenous iron supplementation in orthopedic surgery,^9^ we presumed a clinically meaningful but less optimistic 35% decrease of blood transfusions and/or postoperative nosocomial infection in the active treatment group. The primary end point (a composite of RBC or postoperative nosocomial infection) was assumed in the control group to be 20.5% and in the active treatment arm 13.3%. Therefore, patients needed per group with alpha 5% 2-sided and beta 0.80 was 96. With a dropout rate of 5%, we planned to enroll a total of 210 patients. With the protocol amendment, when patients with other types of cardiac surgery, including valvular heart disease and ascending aortic surgery were also included, power calculations were redone, but the required sample size remained the same.

### Postoperative Infection Definition

Postoperative infection was defined by the Centers for Disease Control and Prevention protocol (https://www.cdc.gov/hai/ssi/ssi.html) and surgeons were trained before study commencement on the criteria to achieve uniform reporting of infections. Wound infection was defined as clinical signs of infection (fever, swelling, redness, new or worsening pain, secretion from wound, or dehiscence of wound without trauma) and a rise in infection parameters (eg, C-reactive protein or white blood cell count) without other explanatory diagnosis (eg, postpericardiotomy syndrome). Any abscess in the operated area was considered a wound infection. Mediastinitis is defined as a wound infection (see above) involving the sternum and or mediastinum or clinically suspected mediastinitis that was operatively treated. Pneumonia was defined as clinical signs of lower respiratory tract infection and fever and rise in infection parameters and imaging finding suggestive of pneumonia on chest radiography or computed tomography.

### Data Monitoring Committee

The independent data monitoring committee consisted of a cardiac surgeon, clinical pharmacologist, hematologist, and biostatistician. The duty of the committee was to protect the ethical and safety interest of patients and all others who may possibly be exposed to the study medication. The committee reviewed ongoing end point and safety data (ie adverse events and serious adverse events) in an unblinded way and made recommendations to the Investigator regarding study modification and/or termination. There were no safety issues in the interim analyses at 50 patients; however, based on the committee statistician's assessment, the study was recommended to be discontinued on a futility basis with regard to reaching the primary end point reduction of RBC >2 U rates and nosocomial infections.
